# Assessing the Performance of BioEmu in Understanding Protein Dynamics

**DOI:** 10.3390/ijms27062896

**Published:** 2026-03-23

**Authors:** Jinyin Zha, Nuan Li, Mingyu Li, Xinyi Liu, Ruidi Zhu, Li Feng, Xuefeng Lu, Jian Zhang

**Affiliations:** Department of Pharmaceutical and Artificial-Intelligence Sciences, Institute of Medical Artificial Intelligence, Shanghai Jiao Tong University School of Medicine, Shanghai 200025, China

**Keywords:** conformational ensemble, deep generative model, Boltzmann distribution, ensemble docking, mutation

## Abstract

Understanding the dynamic conformations of proteins is important for rational drug discovery. While molecular dynamics (MD) simulation is the primary tool for this purpose, it is both resource- and time-consuming. Recent advances in deep learning offer an attractive alternative by generating conformational ensembles directly from protein sequences. However, the scope of applying such models to protein dynamics studies remains underexplored. Here, we tested the performance of a representative model, BioEmu, across several tasks related to protein dynamics. Our results show that BioEmu can not only generate multiple conformations but also effectively reproduce fundamental properties including residue flexibility, motion correlations, and local residue contacts. However, it fails to predict a mutation-induced shift in conformational distribution and exhibits a preference for higher-energy conformations over lower-energy ones in some cases, indicating that it does not reproduce a right Boltzmann-weighted ensemble. Furthermore, the BioEmu-generated conformations provide only limited improvement in ensemble docking. These findings delineate the current capabilities and limitations of sequence-based generative models for conformational sampling. Also, they highlight several directions for future development—that further energy-based fine-tuning is needed for tasks related to conformational distributions and atom-level generative model is required to study the intermolecular relationship.

## 1. Introduction

Understanding dynamic conformations [1,2] of proteins is important for rational drug discovery [3,4]. The conformations could help researchers in comprehending protein functions [5,6,7], identifying cryptic binding sites [8,9,10], and providing structures for ensemble docking [11,12,13]. Experimentally resolving dynamic conformations remains challenging because only few conformations are stable enough to be observed [14]. Even with modern techniques like time-resolved cryo-EM [15], it is still difficult to capture complete protein motions experimentally.

Protein dynamics are more commonly studied by computational methods, particularly molecular dynamics (MD) simulations [16,17,18]. However, MD is in fact very time-consuming. It integrates the system by femtoseconds under Newton’s law, so that simulating a micro- or millisecond protein dynamics would require 10^9^–10^12^ steps of repeated calculations. A series of methods have been developed to accelerate MD simulations [19]. On the one hand, hardware advances mainly focus on parallel computing, including use of graph computing unit (GPU) [20] and developing specialized machines like Anton [21]. Algorithmic improvements include enhanced sampling and coarse-grained (CG) simulations. Enhanced sampling [22,23,24] introduces bias during simulations to reduce the time spent revisiting conformational states but usually requires prior knowledge of protein motions and careful reweighting procedures. CG simulations [25,26,27] save time by calculating a reduced representation of protein with a larger integration step, but they sacrifice accuracy and the CG forcefields often need case-specific refinement [28,29]. Therefore, new methods are still needed to achieve more efficient sampling of protein conformations.

Very recently, several sequence-to-ensemble (seq2ens) methods [30,31,32] have emerged, which take an amino acid sequence as input and directly generate conformational ensembles. These methods are conceptually attractive because they avoid the repetitive calculations of MD and could possibly reduce sampling time from days or weeks into hours. Seq2ens methods could be classified into two groups. The first group modifies inputs of AlphaFold [33,34], for example, using cherry-picked multiple sequence alignments (MSAs) [35,36,37] or templates [38,39]. These attempts are based on the hypothesis that AlphaFold has memorized conformational diversity in Protein Data Bank (PDB) [40] but predicts an averaged structure. Therefore, masking or perturbing inputs can steer predictions toward alternative conformations. However, the overall structure diversity is limited by that of PDB, and perturbations are less controllable, which could lead to unphysical conformations [30]. More crucially, conformations generated do not obey Boltzmann distributions so that energetic information is loss, limiting downstream analysis like identifying key intermediates of conformational transitions [41]. Another group of methods is sequence-conditioned generative models [42,43,44,45,46,47,48]. These models map an easy-to-sample prior distribution onto the complicated distributions of protein conformation, offering better controllability. Nevertheless, due to the lack of MD dataset for model training, these models are usually trained on static PDB structures so that the Boltzmann distribution is still disobeyed and the conformational diversity is also insufficient. These drawbacks have limited the applications of seq2ens methods, which are currently mostly used to generate seed structures for further MD simulations [49,50], rather than direct acceptance.

One promising direction to overcome these obstacles is to pretrain models on static structure databases and then to fine-tune them on MD datasets. A representative example is BioEmu [46], which is pretrained on AlphaFold Database [51] and finetuned on over 200 ms MD data and 500,000 experimental measurements of protein stability. This model is shown to sample multiple kinds of large-scale conformational changes hard to be captured by classical MD. Ensembles generated by BioEmu achieve Boltzmann-like distributions for small proteins and could reproduce experimental observables. Moreover, the speed of Bioemu is much faster than MD, taking less than an hour to generate 1000 samples for a 300-amino-acid protein.

Here, we investigate to what extent BioEmu could take the place of MD in studying protein dynamics. Different from benchmarking in the original study, we here focused on tasks that frequently exist in classical MD analysis including flexibility, motion correlations, residue contacts, mutational effects, conformational bias and ensemble docking. We do not expect that BioEmu could quantitively match MD-generated data or wet-lab experiments, but we are trying to explore whether it could qualitatively reproduce some of the observations.

## 2. Results

### 2.1. BioEmu Generally Re-Emerges the Tendency of Protein Flexibility

**Figure 1:**
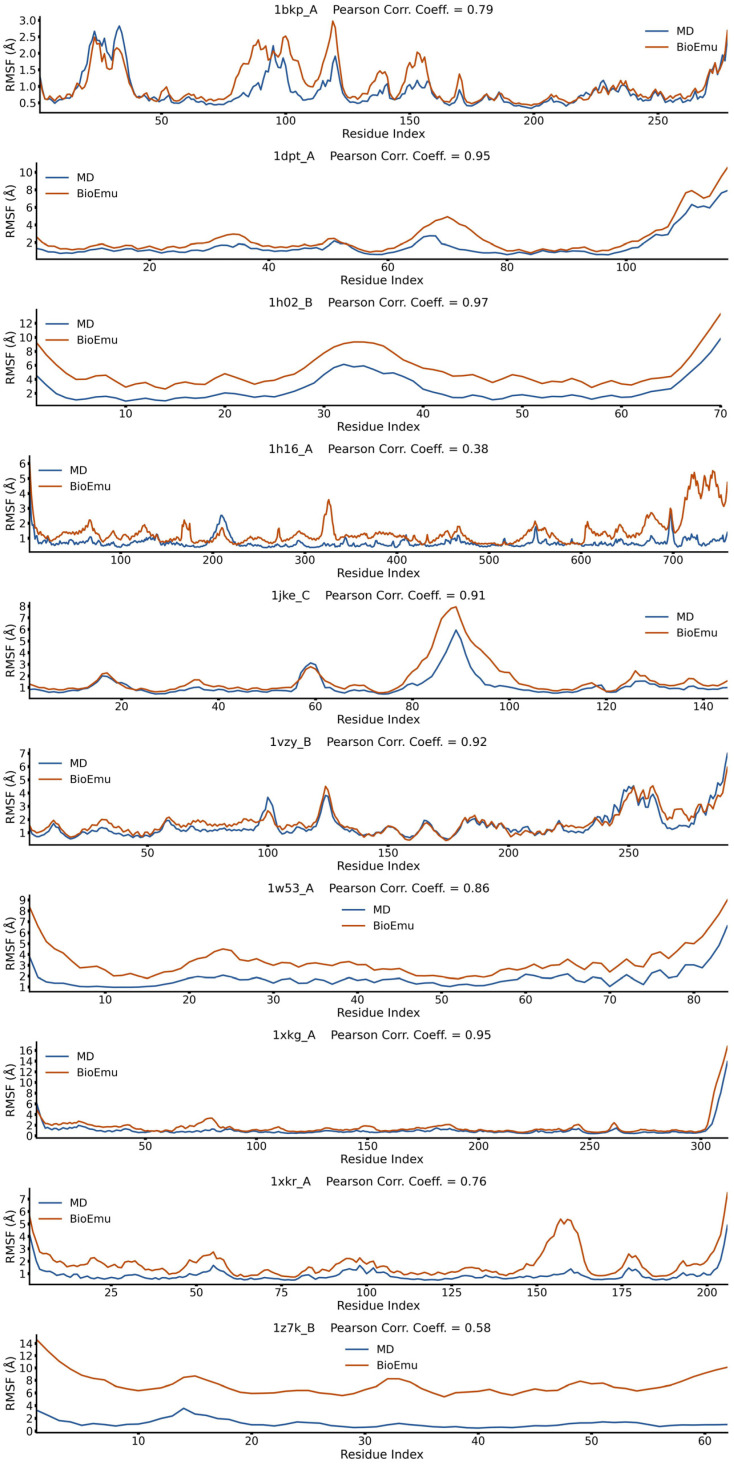
*RMSF* profiles of 1bkp_A, 1dpt_A, 1h02_B, 1h16_A, 1jke_C, 1vzy_B, 1w53_A, 1xkg_A, 1xkr_A and 1z7k_B from BioEmu-generated and MD-generated ensembles. Pearson correlation coefficient describes correlations between the two *RMSF* profiles.

**Figure 2:**
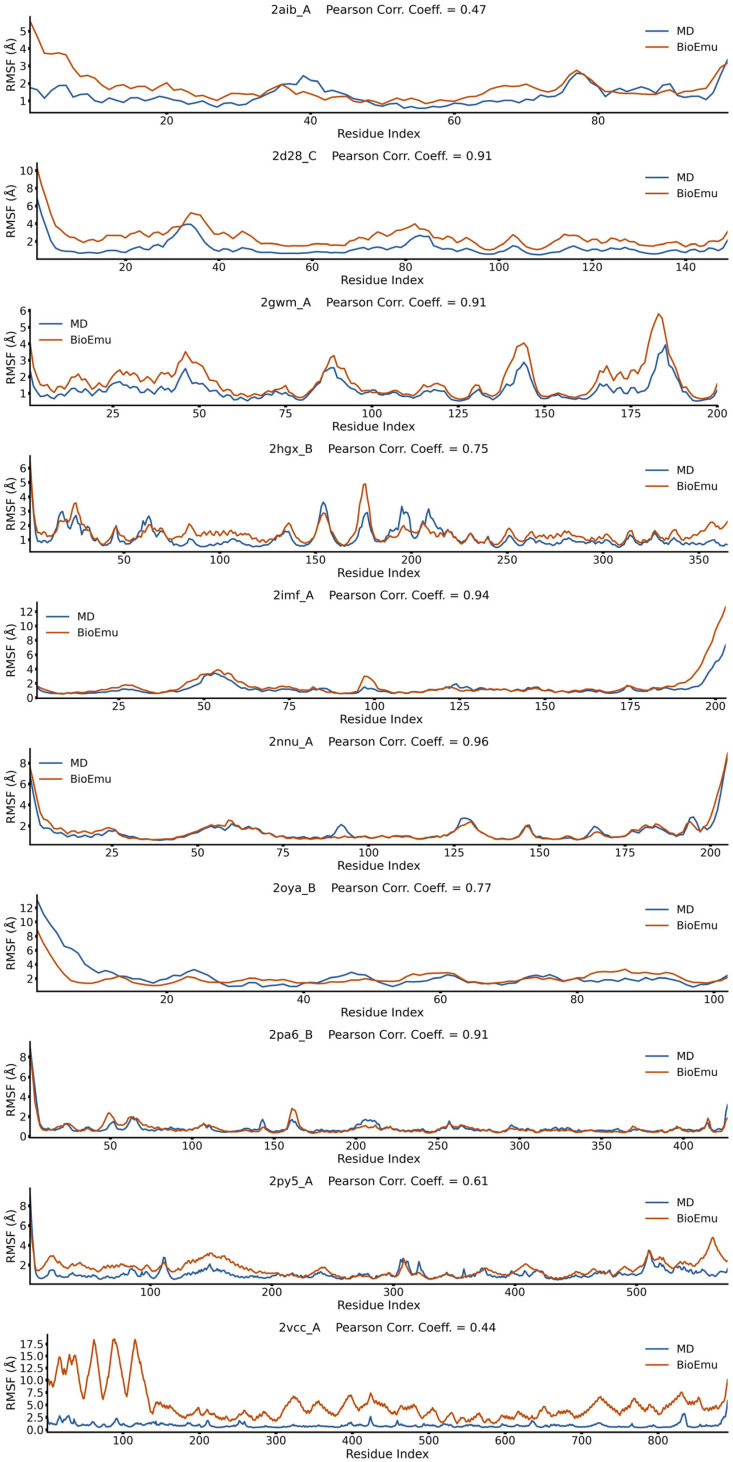
*RMSF* profiles of 2aib_A, 2d28_C, 2gwm_A, 2hgx_B, 2imf_A, 2nnu_A, 2oya_B, 2pa6_B, 2py5_A and 2vcc_A from BioEmu-generated and MD-generated ensembles. Pearson correlation coefficient describes correlations between the two *RMSF* profiles.

**Figure 3:**
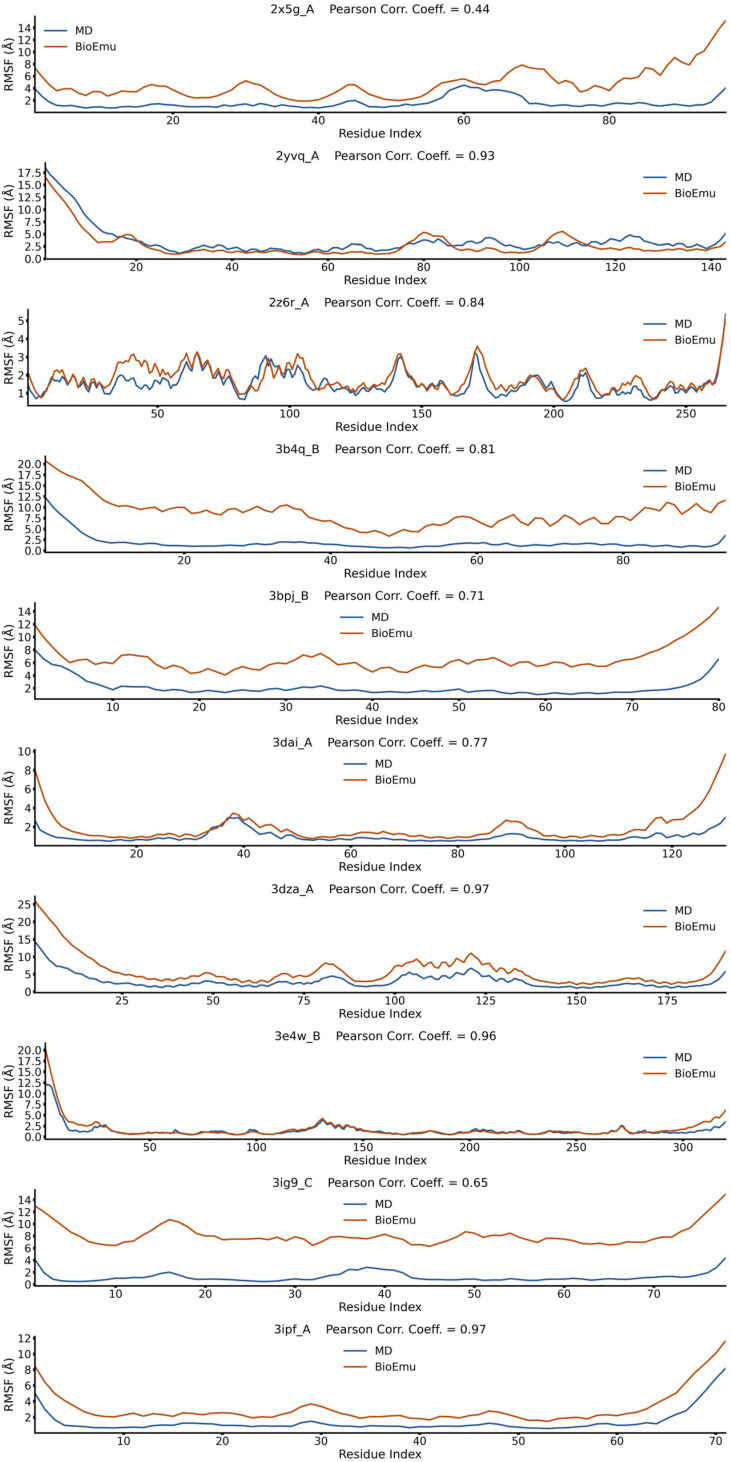
*RMSF* profiles of 2x5g_A, 2yvq_A, 2z6r_A, 3b4q_B, 3bpj_B, 3dai_A, 3dza_A, 3e4w_B, 3ig9_C and 3ipf_A from BioEmu-generated and MD-generated ensembles. Pearson correlation coefficient describes correlations between the two *RMSF* profiles.

**Figure 4:**
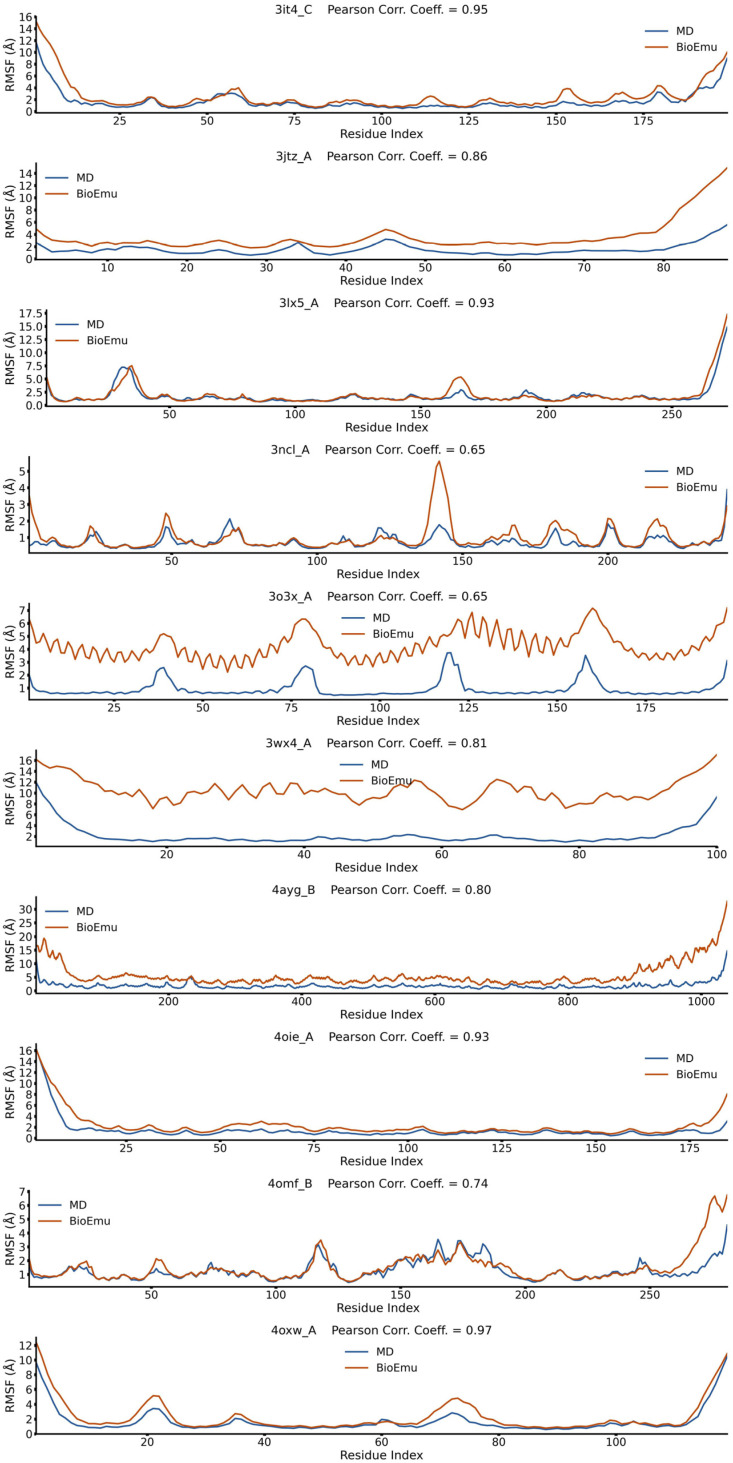
*RMSF* profiles of 3it4_C, 3jtz_A, 3lx5_A, 3ncl_A, 3o3x_A, 3wx4_A, 4ayg_B, 4oie_A, 4omf_B and 4oxw_Afrom BioEmu-generated and MD-generated ensembles. Pearson correlation coefficient describes correlations between the two *RMSF* profiles.

**Figure 5:**
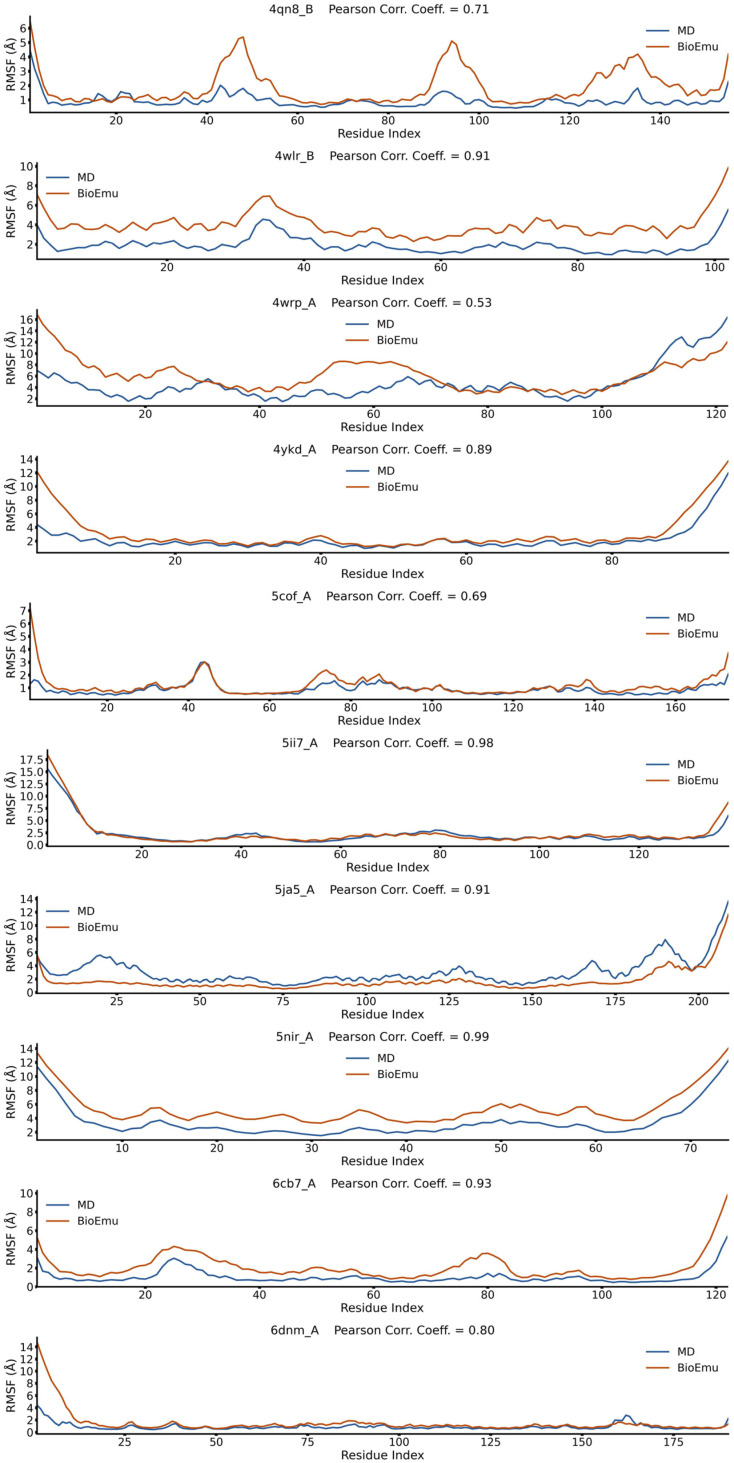
*RMSF* profiles 4qn8_B, 4wlr_B, 4wrp_A, 4ykd_A, 5cof_A, 5ii7_A, 5ja5_A, 5nir_A, 6cb7_A and 6dnm_A from BioEmu-generated and MD-generated ensembles. Pearson correlation coefficient describes correlations between the two *RMSF* profiles.

**Figure 6:**
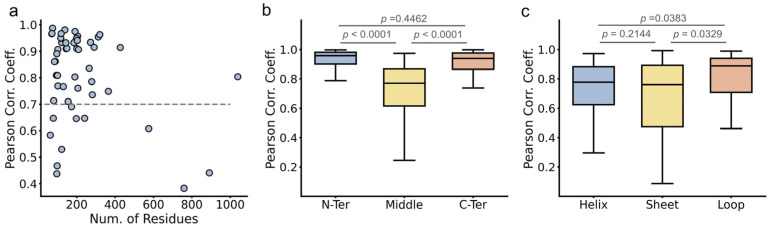
Recovery of *RMSF* by BioEmu. (**a**). Relationship between Pearson correlation coefficient of *RMSF*s and number of residues in protein. Cutoff value of good correlation is set at 0.7 (gray line). (**b**). Distribution of Pearson correlation coefficient in different regions of protein. (**c**). Distribution of Pearson correlation coefficient in different secondary structures. Statistics test done using Mann–Whitney U test (not normal) except for comparing “Helix” and “Sheet” in (**c**), which uses Welch’s *t*-test (normal without uniform variance).

We first evaluated the capability of BioEmu to reproduce fundamental aspects of protein dynamics. For example, identifying flexible regions of protein is important in explaining functional mechanisms [52]. This is usually achieved by calculating the root-mean square fluctuation (*RMSF*) profile of Cα atoms. We randomly selected 50 cases from ATLAS database [53], generated their conformational ensembles by BioEmu [46], and compared the *RMSF*s (Figure 1, Figure 2, Figure 3, Figure 4 and Figure 5) of BioEmu ensemble and MD (300 ns, from ATLAS) ensembles. It should be emphasized that ATLAS is not included in the training set of BioEmu [46]. Since ensembles in ATLAS are usually shorter in timescale than those predicted by BioEmu, we only hope for a consistency of the tendency of *RMSF* profiles, for example, whether both ensembles could identify shared flexible regions (peaks in *RMSF* profile). Therefore, we used Pearson correlation coefficient (*PCC*) instead of absolute error to compare the *RMSF* values. As shown in Figure 6a, 39 of 50 ensembles by BioEmu have shown a good consistency (*PCC* > 0.7) in *RMSF* tendency to MD ensembles. No strong correlation is found between *PCC* and number of residues, although low *PCC* values mainly appear in smaller (<100 residues) and larger (>400 residues) proteins. In high- or middle-*PCC* cases, flexible regions (peaks in *RMSF* profile) in MD ensembles could generally be recaptured in BioEmu ensembles, while in low-*PCC* cases, BioEmu ensembles suggested additional flexible regions. Consistency of flexibility is better in terminal region than middle regions of protein (Figure 6b), and also in loop regions than folded regions (Figure 6c), likely because flexibilities in these regions are more regular (ascending or descending along residue number in terminals and invert-U-shaped in loops) than in other regions.

**Figure 7:**
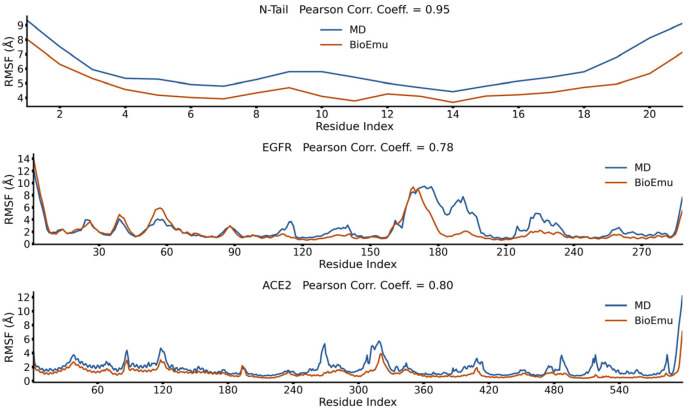
*RMSF* profiles and Pearson correlation coefficient of longer MD trajectories.

We also explored the performance of BioEmu with longer trajectories including N-tail (100 μs) [54], EGFR (22.5 μs) [55] and ACE2 (10 μs) [56]. These trajectories are also not included in the training set of BioEmu. As shown in Figure 7, unlike short trajectories, BioEmu underestimates the overall flexibility. However, it still has a good consistency of *RMSF* tendencies, with *PCC* values all above 0.7. Overall, we concluded here that BioEmu ensembles generally reproduce the tendency of intrinsic flexibility profiles so that it could be utilized to identify flexible regions of a protein. However, it should also be noted that the exact *RMSF* values from BioEmu ensembles could be different from MD trajectories.

### 2.2. BioEmu Generally Managed to Re-Emerge Residue–Residue Correlations

**Figure 8:**
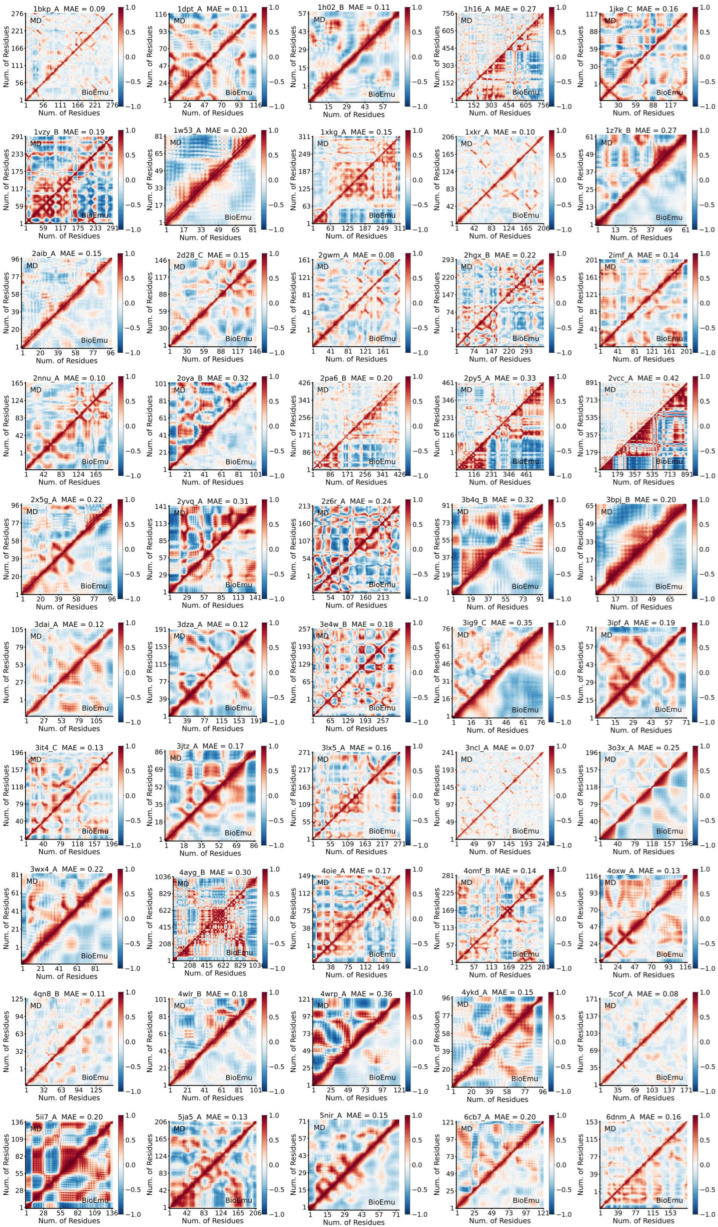
Comparison of combined DCCM from ensembles generated by BioEmu and MD.

**Figure 9:**
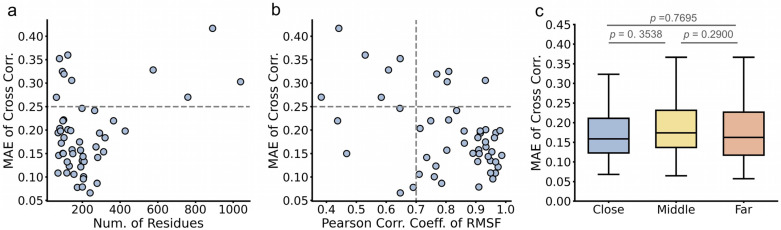
Recovery of DCCM by BioEmu. (**a**). Relationship between *MAE* of cross-correlations and number of residues in protein. Cutoff value of low *MAE* is set at 0.25 (gray line). (**b**). Relationship between *MAE* of cross-correlations and Pearson correlation coefficient of *RMSF*s. Cutoff value of low *MAE* is set at 0.25 and cutoff value of good correlation is set at 0.7 (gray lines). (**c**). Distribution of *MAE* of cross-correlations of residue pairs in different distances. Close: <20% protein length; middle: 20~80% protein length; far: >80% residue length. Statistics test done using Mann–Whitney U test (not normal).

Residue–residue correlation describes the coordinated motions between amino acids. Especially, correlations between distal residues are of particular interest, as they can provide insights into allosteric regulation [6,57,58]. The correlations are usually described by the dynamic cross-correlation matrix (DCCM) [59]. We calculated DCCM of 50 selected cases from ATLAS using BioEmu- and MD-derived ensembles. To facilitate visual comparison between the two matrices for each protein, we generated combined plots where the upper triangle displays the MD DCCM and the lower triangle displays the BioEmu DCCM [60] (Figure 8). The similarity between corresponding DCCM pairs was assessed using the mean absolute error (*MAE*), and the results show that 40 of 50 ensembles achieve an *MAE* below 0.25 (Figure 9a), while most regions of DCCM are well re-emerged (Figure 8). Like *RMSF* in Figure 6a, although no strong relationship is found between *MAE* of DCCM and number of residues, proteins with larger (>400 residues) or smaller (<100) residues are less likely to well-recover cross correlations. It could be found that in poor *MAE* cases, BioEmu ensemble has a much stronger inter-residue motion correlation though this might be reasoned by insufficient MD sampling. Despite with a similar tendency to *RMSF*, we found that cases with poor *RMSF* agreement did not necessarily show poor DCCM agreement, and vice versa. (Figure 9b). A balanced distribution of dots is found in the first (good *RMSF*, poor DCCM), second (poor *RMSF*, poor DCCM) and third (poor *RMSF*, good DCCM) quadrant of Figure 9b. Finally, we found no significant difference in *MAE* among close (<20% protein length), middle (20~80% protein length) and far (>80% residue length) residues (Figure 9c). In summary, these results demonstrate that BioEmu is capable of recapitulating most networks of residue–residue dynamic correlations observed in MD simulations.

### 2.3. BioEmu Could Recover Well Most of the Residue–Residue Contacts

**Figure 10:**
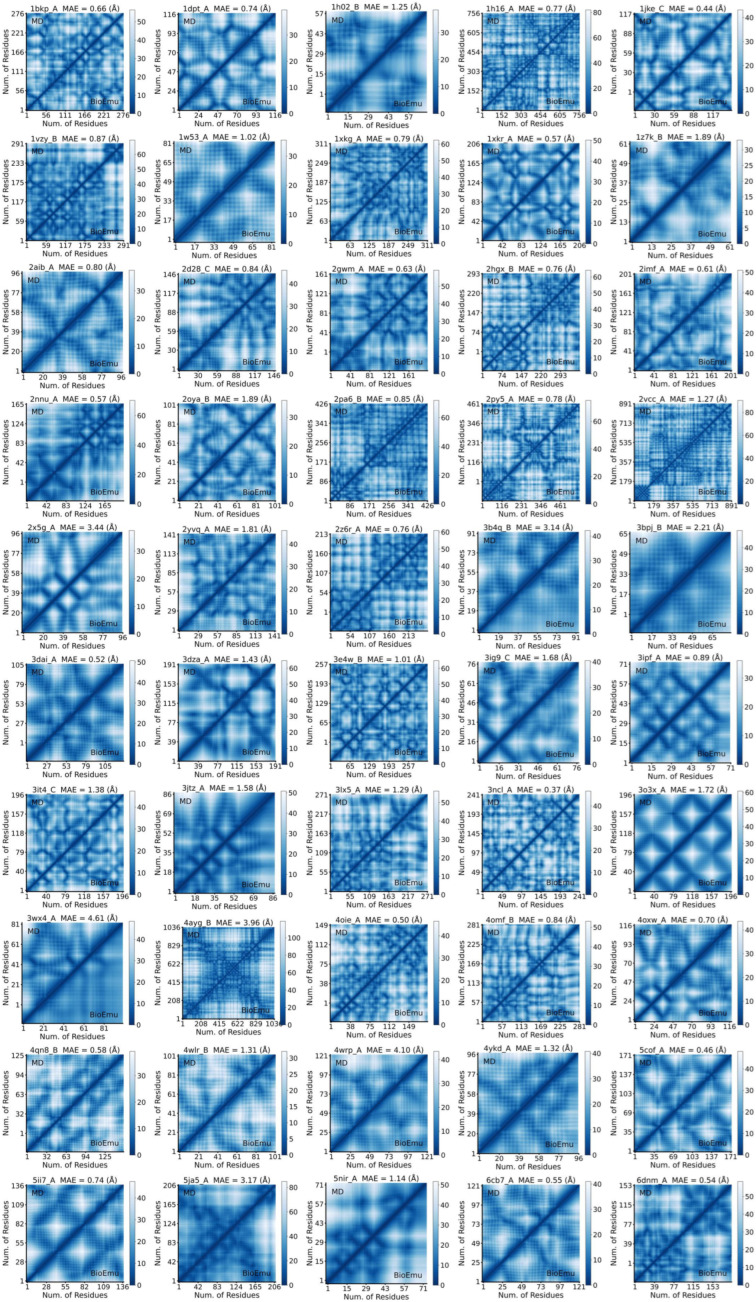
Comparison of combined contact maps from ensembles generated by BioEmu and MD.

**Figure 11:**
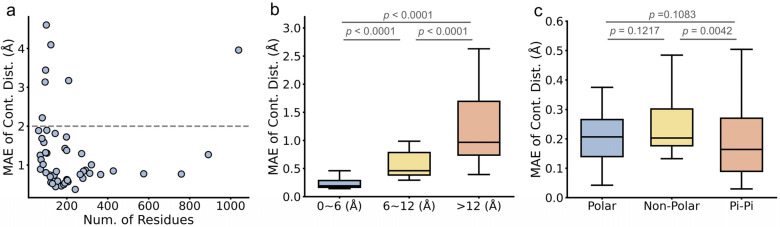
Recovery of contact map by BioEmu. (**a**). Relationship between *MAE* of contact maps and number of residues in protein. Cutoff value of low *MAE* is set at 2 Å (gray line). (**b**). Distribution of *MAE* of contact maps of residue pairs in different distances. (**c**). Distribution of *MAE* of cross-correlations of different kind of interaction pairs. Statistics test done using Mann–Whitney U test (not normal).

As a complementary of residue–residue correlations, which reflects long-range effects, residue–residue contact reflects local interactions like salt bridges and pi-pi stackings. Residue–residue contacts are usually shown by contact map (CM) [61]. Here, we employ a distance-based representation of the contact map, which is a symmetric pair-wise matrix describing the mean smallest distance of two residues along the trajectories. Since BioEmu outputs backbone and Cβ, only the five kinds of atoms are considered in distance calculations. We calculated CM of 50 selected cases from ATLAS using BioEmu and MD ensembles. For visual comparison, the MD-derived CM is displayed in the upper triangle and the BioEmu-derived CM in the lower triangle (Figure 10). Using 2 Å as criteria, where the CMs are generally consistent (Figure 10), it is observed that 43/50 cases have shown a good preservation of CM (Figure 11a). Unlike *RMSF* and DCCM, poorer performance mainly enriches only in smaller proteins. *MAE* of CM is found strongly correlated to the averaged contact distance in MD ensembles, that close residue contacts are found with rather low *MAE* (below 0.5 Å) (Figure 11b). That is important because this is the aim for building CM. Furthermore, we analyzed the preservation of specific interaction types by extracting polar–polar (oppositely charged), non-polar (uncharged), and pi–pi (aromatic) interactions from the set of close contacts. Overall, no significant difference in *MAE* was observed among these interaction categories (Figure 11c), although pi–pi was found to be slightly better preserved than non-polar interactions. Collectively, the above findings indicate that BioEmu could be used to understand residue–residue interactions in protein dynamics.

### 2.4. BioEmu Fails to Distinguish Different Mutation Effects

**Figure 12:**
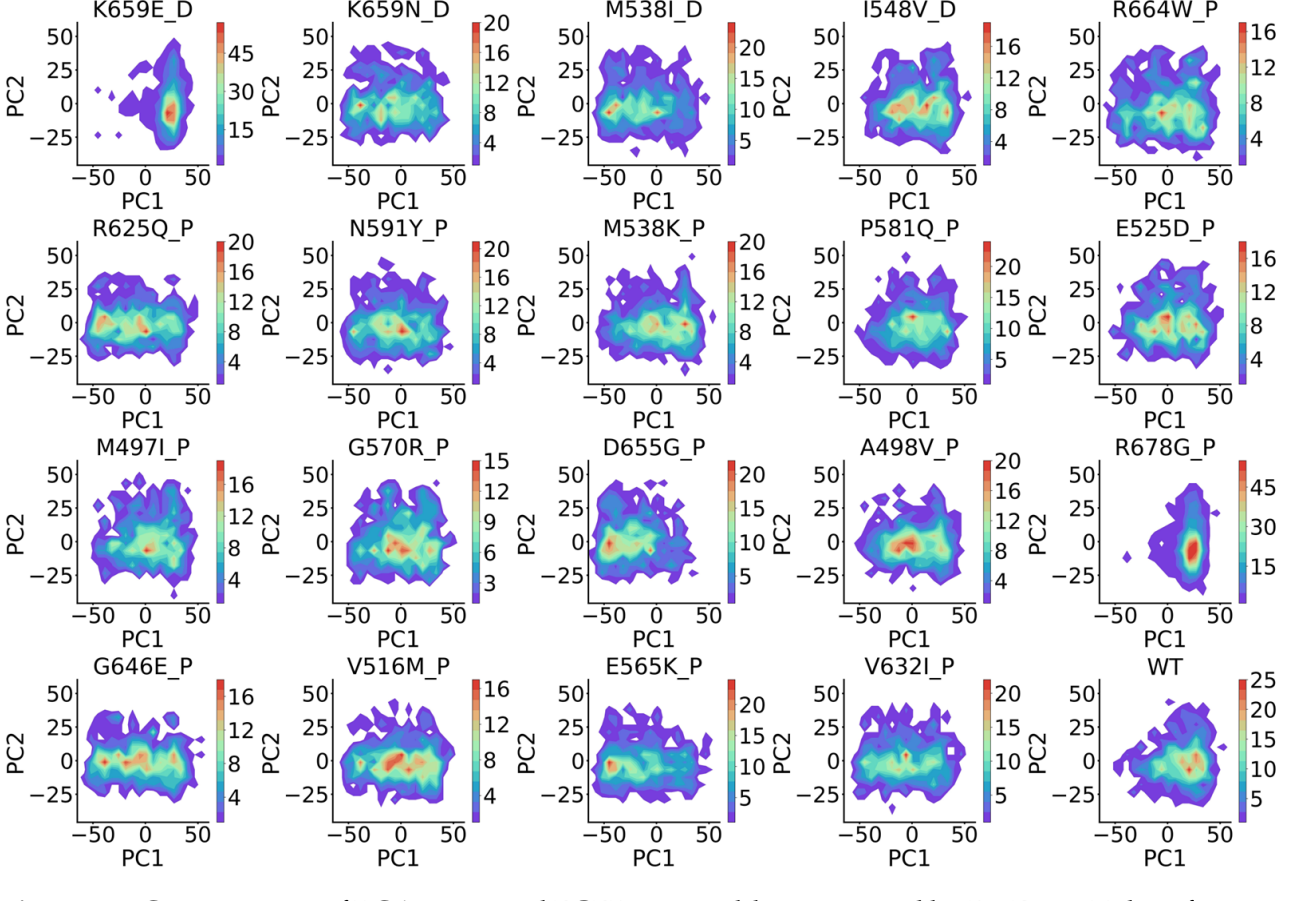
Contour map of PCA-projected FGFR2 ensembles generated by BioEmu. Titles of mutations are split by underline. Before the underline is the position and amino-acid change in the mutation and after the underline is the type (P for passenger and D for driver) of mutations. The color bar describes the local density of conformations. WT is wild type.

**Figure 13:**
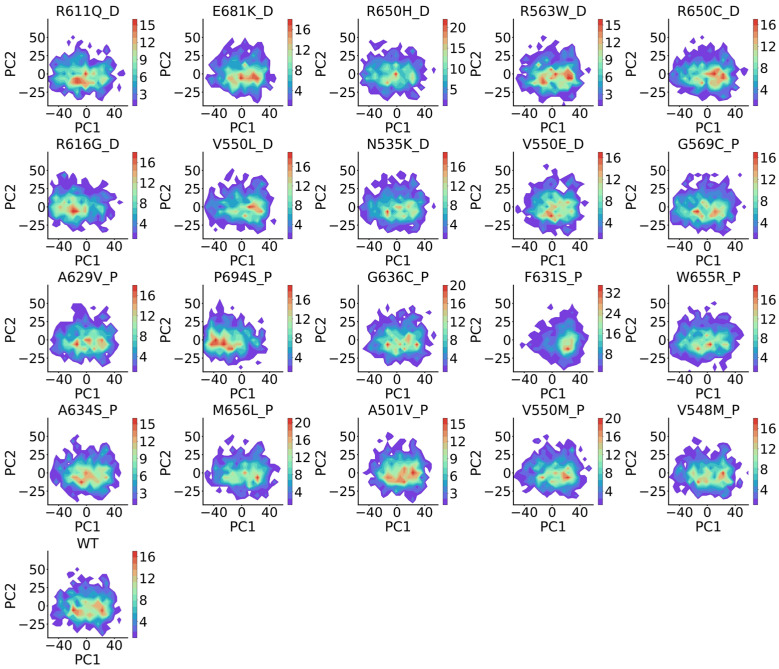
Contour map of PCA-projected FGFR4 ensembles generated by BioEmu. Titles of mutations are split by underline. Before the underline is the position and amino-acid change in the mutation and after the underline is the type (P for passenger and D for driver) of mutations. The color bar describes the local density of conformations. WT is wild type.

**Figure 14:**
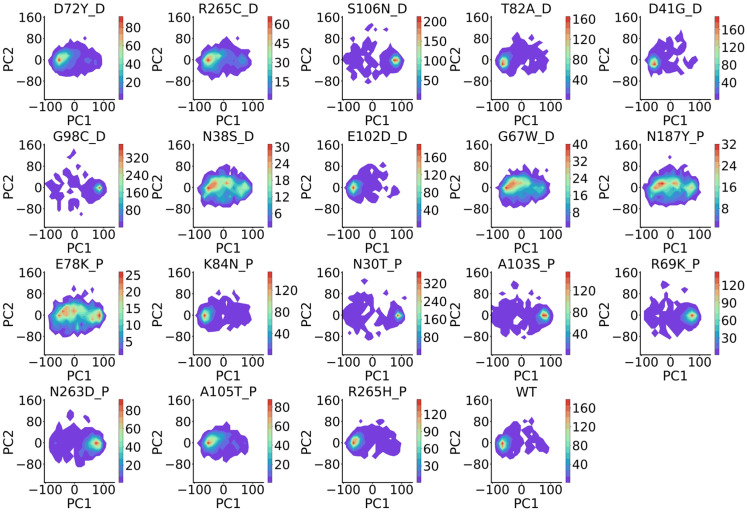
Contour map of PCA-projected MLH1 ensembles generated by BioEmu. Titles of mutations are split by underline. Before the underline is the position and amino-acid change in the mutation and after the underline is the type (P for passenger and D for driver) of mutations. The color bar describes the local density of conformations. WT is wild type.

**Figure 15:**
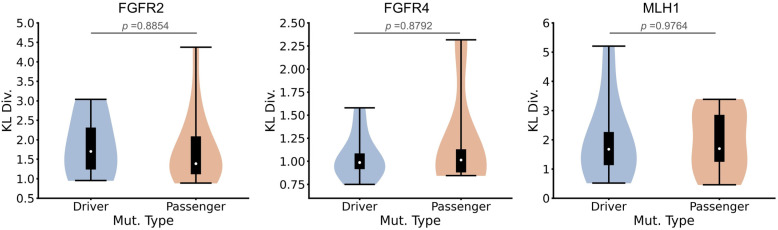
Distribution of KL-divergence of conformational distributions of mutants to WTs in the three proteins. Statistics test done using Student’s *t*-test (normal with uniform variance) for MLH1 and Mann–Whitney U test (not normal) for FGFR2 and FGFR4.

We next explore some more complicated applications. A popular topic in studying protein dynamics is to understand mutation effects [62,63,64]. This is because many disease-causing mutations may not significantly change static structures but would alter the distribution of conformational ensemble [65]. We collected three proteins, all with recorded passenger and driver mutations of cancer [66]. These mutations were not included in the training set of BioEmu. Ideally, ensemble of driver mutations should be more different from the wild-type (WT) ensemble, compared to passenger mutations. We generated ensembles of WT and mutant sequences with BioEmu and projected them to 2D using principal component analysis (PCA) (Figure 12, Figure 13 and Figure 14). It could be observed that the results vary from case to case. In FGFR4, the conformation ensemble is similar among WT, driver mutations and passenger mutations, while in FGFR2 and MLH1, both driver and passenger mutation contain some ensembles resembling WT ensembles and other ensembles different from WT ensembles. These observations could also be quantified in Figure 15 and Tables S2–S4, showing that driver mutations and passenger mutations could not be distinguished based on the difference in conformational distributions (KL divergence) between WT and mutants. For FGFR2 and FGFR4, some passenger mutations even exhibited higher KL divergence from the WT than driver mutations. The above results indicate that BioEmu, in its current form, cannot effectively differentiate driver and passenger mutations. Similar observations is also reported in a recent study of protein engineering [67]. Mutations to protein sequence are more likely to be adding a random noise to the ensemble rather than outputting the real mutation effects. These results are in fact not surprising, because BioEmu [46], as well as the pretrained Evoformer [34], is trained primarily on databases of wild-type protein structures. Although trajectories of mutant proteins are included during the fine-tuning procedures of BioEmu, the training objective of these data mainly focused on folding–unfolding events (i.e., the second fine-tuning stage of protein stability) instead of conformational transitions among folded states.

### 2.5. BioEmu Does Not Exhibit a Bias Towards Physically Stable Conformations

**Figure 16:**
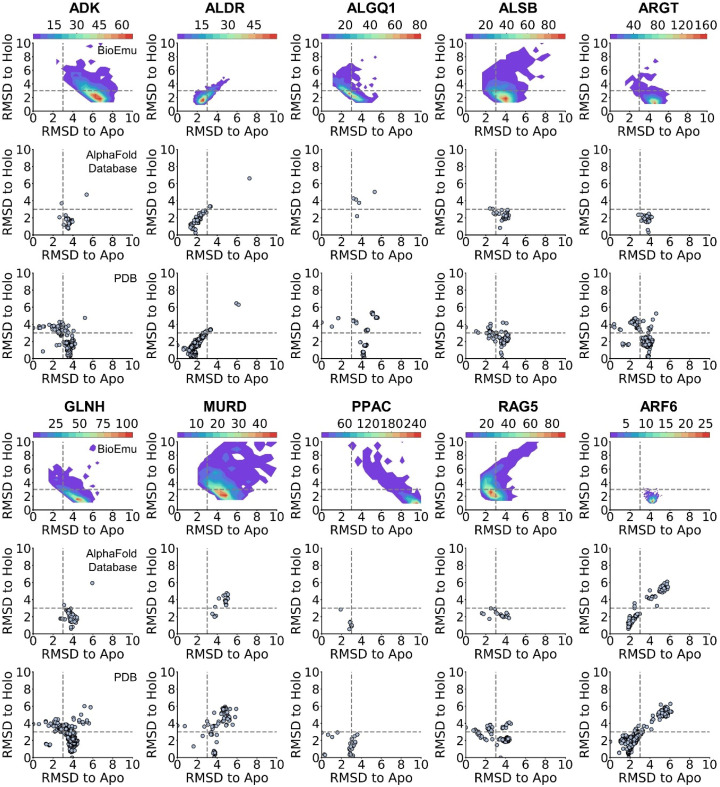
Studying conformational bias by BioEmu. Contour maps or scatter plots of 2-state-*RMSD* (*RMSD* to apo and holo state)-projected ensembles generated by BioEmu, from PDB and AlphaFold Database. Each row represents a method and each column represents a protein. The color bar describes the local density of conformations.

An important reason for requiring seq2ens models to adhere to a Boltzmann distribution is the need to preferentially identify energetically stable conformations for downstream analysis. Namely, models should generate a higher proportion of low-energy conformations and fewer high-energy ones. To test whether BioEmu exhibits such bias, we collected 10 proteins [68] known to undergo conformational changes upon binding their orthosteric ligands. In the absence of ligand, the apo conformation is expected to be more stable for these systems. We used BioEmu to predict ensemble for the 10 proteins. As shown in Figure 16, although trajectories with conformational transitions were not explicitly used during fine-tuning, BioEmu successfully sampled both apo and holo conformations in 9 of 10 cases (except ARF6, using *RMSD* cutoff of 3 Å), possibly thanks to the good pretraining stage on AlphaFold database. However, the conformations generated all biased towards the less stable holo conformation, with the majority of samples falling within the fourth quadrant of an apo-holo *RMSD* comparison plot. To explain the observations, we calculated apo and holo *RMSD*s values of sequentially similar protein structures in PDB [40,68] and AlphaFold Database [51] (Figure 16). The distribution of these experimental and predicted static structures closely aligns with that of BioEmu ensembles, also showing a bias towards holo conformations. Since the first stage of BioEmu training is based on AlphaFold Database, these results suggest that the initial pretraining step exerts a lasting influence on the model’s sampling behavior. Subsequent fine-tuning on MD datasets and experimental stability measurements appears insufficient to steer the model towards an energetically weighted Boltzmann distribution.

### 2.6. BioEmu Has a Minor Improvement in Ensemble Docking

Molecular docking [69,70] is a central technique in virtual screening, which predicts the binding poses as well as affinities of protein–ligand complexes. A well-known limitation of classical docking is its reliance on a single, fixed protein conformation, which neglects the inherent flexibility of binding pockets. As a complement, ensemble docking [11,12,13] is put forward to perform molecular docking on an ensemble of pocket conformations. However, this requires an extensive sampling of pocket conformations [71,72], usually requiring specific sampling techniques like mixed-solvent molecular dynamics [73]. Here, we tested whether BioEmu could be utilized to generate ensemble structures for ensemble docking.

**Figure 17:**
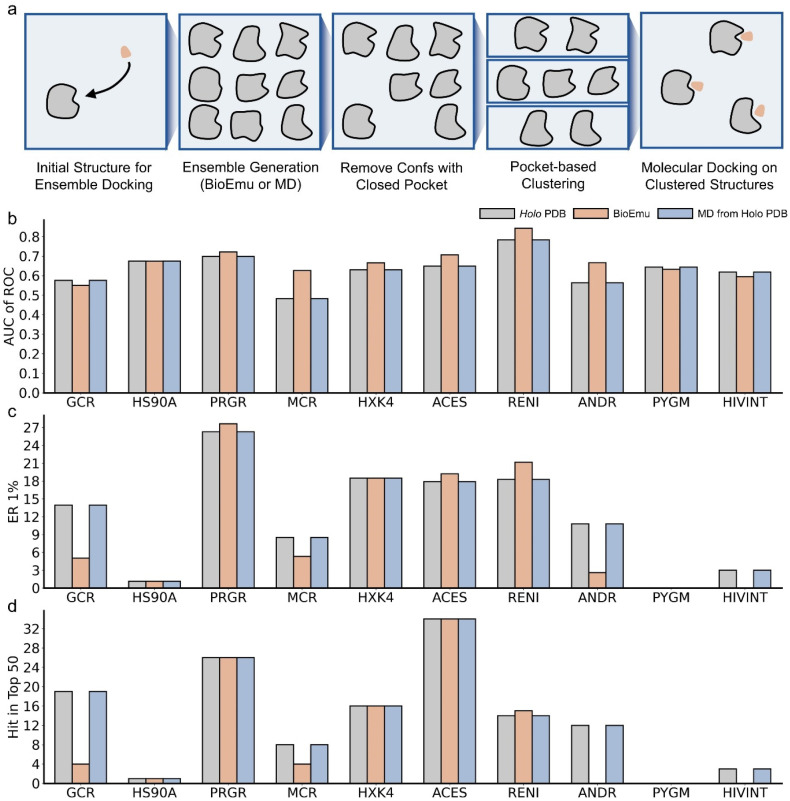
Ensemble docking by BioEmu-generated conformations. (**a**). Workflow of ensemble docking. (**b**). Area under the receiver–operation curve in discriminating active and decoy molecules during ensemble docking the 10 cases. (**c**). Enrichment of active molecules when false-positive rate is 0.01 during ensemble docking the 10 cases. (**d**). Active molecules found in the top 50 after ensemble docking the 10 cases.

In detail, we established the following workflow (Figure 17a). Initially, ensembles are generated via BioEmu or MD. Since BioEmu only predicts coordinates for backbone and Cβ atoms, the full-heavy-atom structures are amended by cg2all [74]. Ensemble conformations are filtered by exposure of the predefined pocket, followed by structural clustering of pocket coordinates. Finally, five clustered structures along with the initial structure (the holo PDB structure for pocket defining) are used for ensemble docking.

We benchmarked the workflow on DUD dataset [75], which contains positive and decoy molecules for various protein targets. We selected ten cases previously reported to benefit from docking against multiple PDB structures [13]. Ensembles were generated by BioEmu and also by three independent 500 ns MD simulations (MD) as well as using the initial holo structure for comparison. The docking performance was evaluated by the discrimination of active and decoy molecules (AUC of ROC) and early enrichment of active compounds (ER 1% and Hit in Top 50) [13]. As shown in Figure 17b–d, ensembles derived from MD do not improve docking in any cases, suggesting that three rounds of 500 ns MD simulation are insufficient to capture novel and ligand-compatible pocket conformations. For BioEmu, six cases with improved AUCs are observed while in three cases the AUCs are declined (Figure 17b). The decline may be attributed to the generation of less physically realistic conformations, potentially due to limitations in BioEmu and in the subsequent all-atom reconstruction. Furthermore, the improvement in AUC does not guarantee a better early enrichment (Figure 17c,d), which might be more important in virtual screening. In fact, only three cases are observed with improved ER 1% and one case with improved Top 50 hits. Conversely, a poorer AUC typically led to poorer enrichment metrics. Based on the results, we concluded that generating ensembles with BioEmu could, to some extent, enlarge the conformations of the binding pocket and improve discrimination between active and decoy molecules. However, no significant improvement was observed in early enrichment measures, so that their utility for enhancing ensemble docking appears limited in this context. The limitations might be attributed to neglect of predicting side-chain coordinates. Reconstructing all-atom structures from coarse-grained representations may accumulate errors that impact the final ensemble-docking performance.

## 3. Discussion

Understanding protein dynamics is crucial for rational drug discovery [1,3,4], yet it remains largely reliant on molecular dynamics (MD) simulations [16,17,18], which is time- and resource-consuming. The recent development of sequence-to-ensemble (seq2ens) models [30,31,32,35,36,37,38,39,42,43,44,45,46,47,48,49,50], which directly generate conformational ensembles from amino acid sequences, offers an attractive alternative. Among these, BioEmu [46] stands out for better performance in sampling multiple conformations, accurate recovery of physical observables and higher sampling speed. In this study, we systematically evaluated the extent to which BioEmu can replace traditional MD simulations in the study of protein dynamics.

Our benchmarking was divided into two categories. The first assessed whether BioEmu could recover fundamental characteristics of protein dynamics, including tendency in residue flexibility, long-range motion correlations, and local residue contacts, especially for proteins within 100 to 400 residues. BioEmu demonstrated strong performance across these basic tasks, indicating that it has effectively learned essential principles of protein conformational dynamics. The second category focused on more complex applications: predicting mutation-induced ensemble shifts, reproducing energetically biased conformational distributions, and generating structures for ensemble docking. BioEmu’s performance was notably weaker in these advanced tasks, which can be attributed to two primary limitations. Firstly, although BioEmu samples a broad range of conformations, it appears to lack an inherent understanding of the underlying physical energetics, i.e., the energy of conformation with a given sequence. Our analysis revealed that despite sampling both apo and holo conformations, BioEmu bias towards the less stable holo conformation for multi-conformation proteins. We further showed that this is possibly an influence from the first-stage training on AlphaFold Database [51]. Additionally, mutations introduced to the sequence often result in unspecific, noise-like shifts in the predicted ensemble rather than biologically meaningful changes, likely due to the scarcity of mutant structures in both the pretraining and fine-tuning datasets. Secondly, BioEmu predicts only the coordinates of backbone and Cβ atoms, rather than full heavy atoms. This coarse-grained representation limits its direct applicability in scenarios where all-atom coordinates are required like ensemble docking. Reconstructing all-atoms from coarse-grained representation would accumulate error.

Our results indicate two possible routes for improving BioEmu and other seq2ens models. Firstly, for applications requiring accurate conformational distributions, case-specific fine-tuning is likely necessary. A promising approach is energy-based fine-tuning, which explicitly incorporates Boltzmann distribution constraints into the model’s loss function [76,77,78]. These strategies could help the model output the energy-informed distributions, possibly fitting seq2ens models for studying conformational bias. Secondly, developing models that natively predict all-atom structural ensembles [42,48] in order to eliminate reconstruction errors and broaden their utility in tasks involving detailed molecular interactions.

To conclude, our benchmarking demonstrates that BioEmu is an efficient tool to predict multiple protein conformations and to reveal basic dynamic properties like tendency of flexibility, motion correlations, and residue contacts. Its usage in conformational distributions and intermolecular interactions would require careful methodological refinement beforehand. We are aware that the current assessment could be limited, either for using limited case or using 300 ns simulation from ATLAS as ground truth, which might be insufficient to capture slow motions in protein dynamics. Our analysis has shown that the flexibility predicted by BioEmu-generated ensemble could be larger than shorter trajectories (300 ns) but smaller than longer (above 5 μs) trajectories. This is possibly because BioEmu is trained on middle-level (~1 μs) trajectories. Further benchmarking analysis could focus on larger and well-designed datasets.

## 4. Material and Methods

### 4.1. Conformational Sampling with BioEmu

BioEmu [46] generates coarse-grained (backbone and Cβ) protein ensembles from their amino-acid sequences. It uses the pretrained Evoformer module from AlphaFold2 [34] to generate single and paired representations, followed by a diffusion module to predict atomic coordinates. In the current study, we used standard settings of BioEmu to generate 1000 samples for each protein. We maintained the filtering process in BioEmu to remove undesired conformations, so that the final scale of the ensemble is usually below 1000. Sampled conformations were aligned to a reference structure using the Kabsch algorithm [79] to eliminate translational and rotational degrees of freedom.

### 4.2. ATLAS Dataset

Protein flexibility, motion correlations and residue contacts are benchmarked on a random selection of 50 cases (Table S1) from the ATLAS database [53] (https://www.dsimb.inserm.fr/ATLAS (accessed on 5 June 2025)), which contains MD trajectories of 1390 structurally diverse protein chains. Each protein was simulated by 100 ns × 3 rounds, with 1000 frames recorded per trajectory. For each protein, we combined the 3 trajectories to yield a 3000-frame ensemble.

### 4.3. Root Mean Square Fluctuation (RMSF)

Protein flexibility is evaluated using root mean square fluctuations (*RMSF*s) of Cα atoms, which quantify the deviation of each residue from its average position. For an N-frame ensemble, the *RMSF* of the ith Cα is defined by Equations (1) and (2):

(1) $${RMSF}_{i}=\sqrt{\frac{1}{N_{frame}}\sum_{t=1}^{N_{frame}}{\left(x_{i}^{t}-x_{i}^{mean}\right)}^{2}}$$

(2) $$x_{i}^{mean}=\frac{1}{N_{frame}}\sum_{t=1}^{N_{frame}}x_{i}^{t}$$

where $x_{i}^{t}$ is the coordinate of the ith Cα in the tth frame of the ensemble, and $x_{i}^{mean}$ is the average coordinate of the ith Cα along the ensemble. $N_{frame}$ is the number of frames in the ensemble. Prior to *RMSF* calculation, each ensemble was aligned to the initial PDB structure provided in the ATLAS dataset [53].

The similarity between *RMSF* profiles derived from MD and BioEmu ensembles was evaluated using the Pearson correlation coefficient (*PCC*), as defined in Equation (3):

(3) $$PCC=\frac{\sum_{i=1}^{N_{atom}}\left(\left({RMSF}_{i}^{BioEmu}-\bar{{RMSF}_{i}^{BioEmu}}\right)\cdot \left({RMSF}_{i}^{MD}-\bar{{RMSF}_{i}^{MD}}\right)\right)}{\sqrt{\sum_{i=1}^{N_{atom}}{\left({RMSF}_{i}^{BioEmu}-\bar{{RMSF}_{i}^{BioEmu}}\right)}^{2}}\cdot \sqrt{\sum_{i=1}^{N_{atom}}{\left({RMSF}_{i}^{BioEmu}-\bar{{RMSF}_{i}^{BioEmu}}\right)}^{2}}}$$

where ${RMSF}_{i}^{BioEmu}$, ${RMSF}_{i}^{MD}$ denote the *RMSF* of the ith Cα atom in BioEmu and MD ensembles and $\bar{{RMSF}_{i}^{BioEmu}}$ and $\bar{{RMSF}_{i}^{MD}}$ represent the corresponding average *RMSF*s. $N_{atom}$ is the number of Cα atoms.

### 4.4. Dynamics Cross-Correlation Matrix (DCCM)

Motion correlations are evaluated by the dynamics cross-correlation matrix (DCCM) [59]. This is a $N_{res}\times N_{res}$ symmetric matrix (C) describing the motion correlations among residues. Its element $C\left(i,j\right)$ is defined by Equations (4) and (5):

(4) $$C\left(i,j\right)=\frac{\sum_{t=1}^{N_{frame}}{∆x}_{i}^{t}\cdot {∆x}_{j}^{t}}{\sqrt{\sum_{t=1}^{N_{frame}}{\left|{∆x}_{i}^{t}\right|}^{2}\cdot \sum_{t=1}^{N_{frame}}{\left|{∆x}_{j}^{t}\right|}^{2}}}\;$$

(5) $${∆x}_{j}^{t}=x_{i}^{t}-x_{i}^{mean}$$

where ${∆x}_{i}^{t}$ is the displacement of the ith Cα in the tth frame of the ensemble to its average position. To compare DCCMs from MD and BioEmu, we apply mean absolute error (*MAE*), as shown in Equation (6), for a given matrix M:

(6) $$MAE=mean\left(\left|M-M_{ref}\right|\right)$$

### 4.5. Contact Map

Contact map [61] is calculated in a distance-based fashion. It is also an $N_{res}\times N_{res}$ symmetric matrix (D), whose element $D\left(i,j\right)$ describes the mean shortest distance between the ith and jth residues along the ensemble. Since BioEmu outputs only backbone and Cβ atoms, distances were computed considering only these atom types.

### 4.6. Benchmarking of Mutational Effects

We extracted passenger/driver mutations on three proteins, including FGFR2 (P21802, 481-770), FGFR4 (P22455, 467-755) and MLH1 (P40692, 1-336) from the training set of DeepAlloDriver [66]. For each protein, conformational ensembles of wild type (WT) and mutants (Mut) were predicted from their sequences by BioEmu. Principal component analysis (PCA) [79] was done on the combined trajectory (Cα coordinates only), and each individual ensembles were projected to the first and second principal component (PC1 and PC2). To evaluate the difference between WT and mutant ensembles, 2D histograms with identical bin sizes were constructed for all projected ensembles. The difference between the WT and mutant conformational distributions was quantified using the Kullback–Leibler divergence (KL divergence) [80], as defined in Equation (7):

(7) $$KLDiv(WT|\left|Mut\right)=\sum_{i}B_{i}^{WT}\cdot \log\left(\frac{B_{i}^{WT}}{B_{i}^{Mut}}\right)$$

where $B_{i}^{WT}$ and $B_{i}^{Mut}$ describe the ratio of conformations in the ith bin from the MT and the mutant ensemble.

### 4.7. Benchmarking of Conformational Bias

We selected 10 proteins with great conformation changes in their apo and holo state, as shown in Table S5. Apo and holo structures were first retrieved from Protein Data Bank (PDB) [40,68]. Redundant chains and residues were removed to ensure equivalent protein segments were compared. BioEmu was then used to predict a conformational ensemble from the sequence. To assess the bias of generated conformations, we calculated the Cα root mean square distance (*RMSD*) of each conformation to the apo and holo structure as Equation (8):

(8) $${RMSD}^{t}=\sqrt{\frac{1}{N_{atom}}\sum_{i=1}^{N_{atom}}{\left(x_{i}^{t}-x_{i}^{ref}\right)}^{2}}$$

where $x_{i}^{t}$ is the coordinate of the ith Cα in the tth frame of the ensemble, and $x_{i}^{ref}$ is the coordinate of the ith Cα in the reference (apo or holo) structure. Note that the coordinates should be aligned to the reference structure before *RMSD* calculation.

As a comparison, we also calculated the conformational bias on PDB and AlphaFold Database. For PDB, BLAST (version: 2.17.0) in PDB website [81] was used to identify sequentially similar protein chains (e-value cutoff = 0.1, identity > 0%). For AlphaFold Database [51], we extracted UniProt IDs of similar proteins in PDB and fetched relative structures. *RMSD*s of each structure to the apo and holo structure were calculated with TMAlign (version: 20220412) [82].

### 4.8. Benchmarking of Ensemble Docking

We selected 10 cases from the DUD dataset [75] (excluding membrane proteins), which contains positive and decoy ligands of protein targets. BioEmu was used to generate conformational ensembles. Full-atom structures were reconstructed for each frame using cg2all [74]. The all-atom ensemble was aligned to the reference complex structure from DUD. Pocket exposures were then analyzed for each frame as below. AutoSite [83] was employed to detect pockets of a frame, represented by clusters of grid points. Spatial overlaps between each pocket grids and reference ligand (from DUD) were calculated, namely, counting the ratio of ligand atoms which are within 1 Å of any grids. The highest overlap value among all pockets in a frame was recorded as its pocket exposure score. We use a cutoff value of pocket exposure to filter out frames less suitable for docking. Although a larger cutoff value could increase the quality of pocket conformations, it could also decrease the diversity. Therefore, we start to set the cutoff value at 0.5, which is reported by previous study as the median value observed in numerous simulations [84]. However, if no more than 100 frames are retained, the cutoff values decrease iteratively by 0.1 until the requirement is met. Final cutoff values are shown in Table S6. After filtering, the remaining coordinates of pocket atoms (defined as protein heavy atoms within 1 Å of the reference ligand) were extracted and subjected to PCA for dimensionality reduction. The reduced representations were clustered into 5 groups with K-Means [85]. For each cluster, we chose the full-atom conformation with the most favorable AutoSite energy as a representative structure, along with the initial reference structure, for ensemble docking.

Docking of each structure was done with Glide [86,87]. Protein structures were prepared by adding hydrogen atoms and applying minimization. Ligands were prepared using standard protocols. The docking grid was centered on the reference ligand, with an inner box of 6 Å and an outer box of 26 Å. Docking was carried out using Standard Precision (SP) mode. Results were ranked based on the Glide SP score.

Docking performances were evaluated by area under receiver operating curve (AUC of ROC), the enrichment factor at a 1% false-positive rate (ER 1%) and hits in top 50 rankings [13]. The ROC curve was plotted with the true-positive rate (TPR) against the false-positive rate (FPR) at varying Glide SP score thresholds. ER 1% is defined as the ratio of TPR and FPR when FPR is 0.01.

For comparison, we also performed ensemble docking from conformations by MD simulations. In brief, PDB structures were redownloaded from Protein Data Bank [40] and redundant chains were removed. Using the *tleap* module in the AMBER20 suite [88], missing atoms were added, and the system was solvated in a TIP3P water box with a 12 Å margin and neutralized with Na^+^ or Cl^−^ ions. The protein was described with the ff14SB force field. The system was energy-minimized, gradually heated to 300 K, and equilibrated for 100 ps each in the NVT and NPT ensembles. Production simulations were run in the NPT ensemble for 500 ns with GPU accelerated *pmemd*. An integration timestep of 2 fs was used with SHAKE constraints [89] on bonds involving hydrogen atoms. Temperature was regulated using a Langevin thermostat [90] with a collision frequency of 2 ps^−1^. Each system was simulated individually for three rounds.

## Data Availability

Protein structures are retrieved from Protein Data Bank (https://www.rcsb.org/) and AlphaFold Database (https://alphafold.com/). ATLAS data could be acquired from https://www.dsimb.inserm.fr/ATLAS (accessed on 5 June 2025). Dataset for ensemble docking is retrieved from DUD dataset (https://dude.docking.org/).

## Supplementary Materials

The following supporting information can be downloaded at: https://www.mdpi.com/article/10.3390/ijms27062896/s1.
