# Supplementary material for: Assessing the Performance of BioEmu in Understanding Protein Dynamics
Source: Int J Mol Sci. 2026 Mar 23;27(6):2896. doi: 10.3390/ijms27062896 (PMC13026764; doi:10.3390/ijms27062896)
Supplement: Supplementary file 1 [file ijms-27-02896-s001.zip › ijms-4145330-supplementary.pdf]

Supplementary Information

Assessing the Performance of BioEmu in Understanding Protein Dynamics

Jinyin Zha[1], Nuan Li[1], Mingyu Li[1], Xinyi Liu[1], Ruidi Zhu[1], Li Feng[1*], Xuefeng Lu[1*], Jian Zhang[1*]

Affiliations:

[1] Department of Pharmaceutical and Artificial-Intelligence Sciences, Institute of Medical Artificial Intelligence, Shanghai Jiao Tong University School of Medicine, Shanghai, China

*Corresponding author. Email: jian.zhang@sjtu.edu.cn

Supplementary Tables

Table S1. Benchmarking cases from ATLAS.

PDB ID	Chain ID	PDB ID	Chain ID	PDB ID	Chain ID	PDB ID	Chain ID	PDB ID	Chain ID
5COF	A	1VZY	B	1XKG	A	3JTZ	A	3B4Q	B
1H02	B	6CB7	A	1H16	A	1Z7K	B	3O3X	A
1JKE	C	4WLR	B	4OMF	B	1BKP	A	5NIR	A
1XKR	A	2OYA	B	3IPF	A	1DPT	A	2NNU	A
4YKD	A	2Z6R	A	2GWM	A	2PY5	A	4QN8	B

Table S2. Mutations and KL-divergence of conformations to wild-type in FGFR2. (4 driver mutations and 15 passenger mutations)

Wild-Type Residue	Residue ID	Mutant Residue	Mutation Type	KLD
K	659	E	Driver	3.039481
K	659	N	Driver	1.332512
M	538	I	Driver	2.071544
I	548	V	Driver	0.954341
R	664	W	Passenger	1.100256
R	625	Q	Passenger	2.185417
N	591	Y	Passenger	1.580154
M	538	K	Passenger	1.069436
P	581	Q	Passenger	1.057258
E	525	D	Passenger	1.130252
M	497	I	Passenger	0.887802
G	570	R	Passenger	1.257326
D	655	G	Passenger	4.376401
A	498	V	Passenger	1.290628
R	678	G	Passenger	3.039481
G	646	E	Passenger	1.332512
V	516	M	Passenger	2.071544
E	565	K	Passenger	0.954341
V	632	I	Passenger	1.100256

Table S3. Mutations and KL-divergence of conformations to wild-type in FGFR4. (9 driver mutations and 11 passenger mutations)

Wild-Type Residue	Residue ID	Mutant Residue	Mutation Type	KLD
R	611	Q	Driver	1.308399
E	681	K	Driver	0.859333
R	650	H	Driver	1.086012
R	563	W	Driver	0.914759
R	650	C	Driver	0.750049
R	616	G	Driver	1.579494
V	550	L	Driver	0.91668
N	535	K	Driver	0.987453
V	550	E	Driver	1.007674
G	569	C	Passenger	0.87786
A	629	V	Passenger	0.858452
P	694	S	Passenger	2.317854
G	636	C	Passenger	1.014078
F	631	S	Passenger	2.30265
W	655	R	Passenger	0.882143
A	634	S	Passenger	0.845185
M	656	L	Passenger	1.019694
A	501	V	Passenger	1.243213
V	550	M	Passenger	0.914934
V	548	M	Passenger	1.013782

Table S4. Mutations and KL-divergence of conformations to wild-type in MLH1. (9 driver mutations and 9 passenger mutations)

Wild-Type Residue	Residue ID	Mutant Residue	Mutation Type	KLD
D	72	Y	Driver	1.202291
R	265	C	Driver	1.132578
S	106	N	Driver	3.357566
T	82	A	Driver	0.538505
D	41	G	Driver	1.67834
G	98	C	Driver	5.204046
N	38	S	Driver	1.825874
E	102	D	Driver	0.52082
G	67	W	Driver	2.269155
N	187	Y	Passenger	1.436043
E	78	K	Passenger	1.699872
K	84	N	Passenger	0.461494
N	30	T	Passenger	3.387853
A	103	S	Passenger	2.854143
R	69	K	Passenger	3.283634
N	263	D	Passenger	2.717033
A	105	T	Passenger	1.240371
R	265	H	Passenger	0.478708

Table S5. Cases for benchmarking conformational bias.

Name	Full Name	*Apo* PDB	*Holo* PDB
GLNH	Glutamine binding protein	1GGG	1WDN
ARF6	ADP-ribosylation factor 6	1E0S	2J5X
RAG5	Hexokinase	3O80	3O8M
MURD	UDP-N-acetylmuramoylalanine:D-glutamate ligase	1E0D	3UAG
ADK	Adenylate kinase	4AKE	1AKE
ALGQ1	Alginate-binding protein	1Y3Q	1Y3N
PPAC	Manganese-dependent inorganic pyrophosphatase	1K23	1WPM
ARGT	Lysine/arginine/ornithine-binding periplasmic protein	2LAO	1LAH
ALSB	D-allose-binding periplasmic protein	1GUD	1RPJ
ALDR	Aldo-keto reductase family 1 member B1	1XGD	2HV5

Table S6. Cutoff values of pocket exposures.

Case	mcr	prgr	hxk4	pygm	hivint	andr	aces	gcr	reni	hs90a
BioEmu	0.5	0.5	0.3	0.2	0.2	0.5	0.5	0.5	0.5	0.5
MD	0.5	0.5	0.2	0.4	0.4	0.5	0.5	0.4	0.5	0.5
